# The Present Status of Corrosive Sublimate in Surgery

**Published:** 1893-05

**Authors:** 


### The Present Status of Corrosive Sublimate in Surgery.

I want to say a few words on a subject in which we are all interested—the present status of corrosive sublimate in surgery. For a long time all of us who work in general surgery have wondered why we failed to get good results in cases where we thought we had done everything that could be done to assure an antiseptic condition of the part. Two years ago I ceased the use of solutions particularly in fresh wounds, and have lately ceased to use them at all, even in suppurating conditions. Three years ago Welch, of the John Hopkins University, made a report in which he mentioned numerous experiments with sublimate to find out something about the action and power of this drug. He has given us reasons why we oftentimes fail in getting an aseptic condition or having an aseptic outcome of a wound from the use of this substance. It has been proven that the inhibiting power of bichloride of mercury is just so much, which is in proportion to the albuminous material it has to deal with. I think it is proven beyond all doubt by his experiments and by those of his assistants, particularly Abbott, that the present position of bichloride is rather against its use, and that it does more harm than good. If the solution be of sufficient strength to have any effect upon germs already there, it will cause necrosis and prevent healing of the wound, as well as producing a better nidus for the production and operation of other micro-organisms.

I will read from Dr. Abbott's paper his conclusions upon this subject, which may be of interest:

"What conclusions are we justified in drawing from such results as those obtained in the experiments here reported?

"1. It is seen that under the most favorable conditions a given amount of sublimate has the property of rendering inert only a certain number of individual organisms. That is to say, the process is a definite chemical one taking place between the protoplasm of the individual bacteria and the sublimate in solution.

"2. That the disinfecting activity of the sublimate against organisms is profoundly influenced by the proportion of albuminous material contained in the medium in which the bacteria are present.

"3. That the relation between the golden pyogenic staphylococci and sublimate is not a constant one, organism from different sources and of different ages behaving differently when exposed to the same amount of the disinfectant for the same length of time.

"4. That the organisms which survive the exposure to the sublimate may experience a temporary attenuation. This attenuation, however, may be caused to disappear by successive cultivation in normal media.

"5. That by the method employed in these experiments, it is possible to select from a culture the most resistant forms of that culture.

"6. That many of the results of previous experimenters, who have assigned to corrosive sublimate more powerful disinfectant properties against the staphylococcus pyogenes aurens in cultures than the observations reported in this paper indicate, are attributable to the neglect of certain precautions now recognized as essential to the proper conduct of such experiments.

"In the light of these experiments and those of the experimenters quoted in this paper, it is plain that for use in surgical practice the solutions of corrosive sublimate do not possess all the advantages hitherto attributed to them.

"To the employment of sublimate solutions upon wound surfaces it is plain that there exist at least two serious objections:

"First the albumen of the tissues and fluids of the body tend to diminish the strength of, or indeed renders entirely inert, the solution employed.

"And second, the integrity of the tissues is materially injured by the application of solutions of this salt.

"The first objection cannot be met with certainty, for the surgeon possesses no means by which he can determine the albuminous material with which his solutions are to come in contact, and in any case this large amount of albuminous material is an almost insuperable obstacle to complete disinfection with sublimate. He is, therefore, never in a position to say, *a priori*, that his efforts at disinfection of the wound are, or are not, successful.

"The second objection is equally serious. During the past two years we have had sufficient evidence to lead us to believe that the normal tissues and fluids of the body possess the power of rendering inert many kinds of organisms which may have gained access to them. This function is therefore diminished, or indeed may be quite destroyed, by any agent which brings about alterations in the constitutions of these tissues. We know that just such changes as those to which we refer are known to follow the application of sublimate solutions. It is plain that if we bring about in these tissues a condition of superficial necrosis—the condition following upon the application of sublimate—they are much less able to resist the inroads of infectious organisms than they would have been had they been left in their natural condition.

"As a disinfectant, in the strict sense of the word, there are perhaps few substances which possess the property in a higher degree than does corrosive sublimate, but at the same time there is nothing which is employed for this purpose that requires greater care in its manipulation in order to obtain its best results, than does this salt. As we have seen, its action is influenced by a number of conditions which in practical application it is difficult, if not quite impossible, to control.

"For these reasons we seem hardly justified in continuing to give it the first place in the list of substances which may be employed practically for the purpose of rendering harmless materials containing the germs of infectious maladies."

I have found in my practical work, that since I ceased the use of sublimate solutions I have had much more uniformly good results by using simply sterilized water.—Ap. Morgan Vance, M. D., in *Med. and Surg. Reporter.*

